# Pregabalin-associated stuttering and frequent blepharospasm: case report and review.

**DOI:** 10.1007/s40199-020-00354-9

**Published:** 2020-07-06

**Authors:** Lingzhi Ge, Ang Li, Ni Wang, Ping Li, Hongyan Xin, Wenfei Li

**Affiliations:** 1 Department of Dermatology, the First Affiliated Hospital of Shandong First Medical University, Shandong Provincial Qianfoshan Hospital, 16766 Jing-Shi Road, Jinan, 250014 China; 2 Department of Dermatology, the Second Affiliated Hospital of Shandong First Medical University, Tai’an, 271000 China; 3 Department of Orthopaedic Surgery, Shanghai Jiao Tong University Affiliated Sixth, People’s Hospital, 600 Yishan Road, Shanghai, 200233 China; 4 Department of Dermatology, Jimo District People’s Hospital, Qingdao, 266200 China; 5 Department of Surgery, Shandong Chest Hospital, Jinan, 250013 China

**Keywords:** Pregabalin, Side effects, Stuttering, Frequent blepharospasm

## Abstract

Herpes zoster is an acute, painful, herpes skin disease caused by varicella-zoster virus, which may cause viral meningitis. Pregabalin has been shown to be efficacious in the treatment of pain in patients with herpes zoster. However, it has the side effects of neurotoxicity. We describe a 68-year-old female patient with herpes zoster, and she was treated with pregabalin. The patient presented with stuttering and frequent blepharospasm after 3 days of pregabalin treatment. Pregabalin was discontinued, the symptoms of stuttering and frequent blepharospasm completely resolved without any special treatment after one week. In this case, the etiology of stuttering and frequent blepharospasm may be related to pregabalin. Clinicians should be alert to the rare symptoms associated with the use of pregabalin.

**Graphical abstract:**
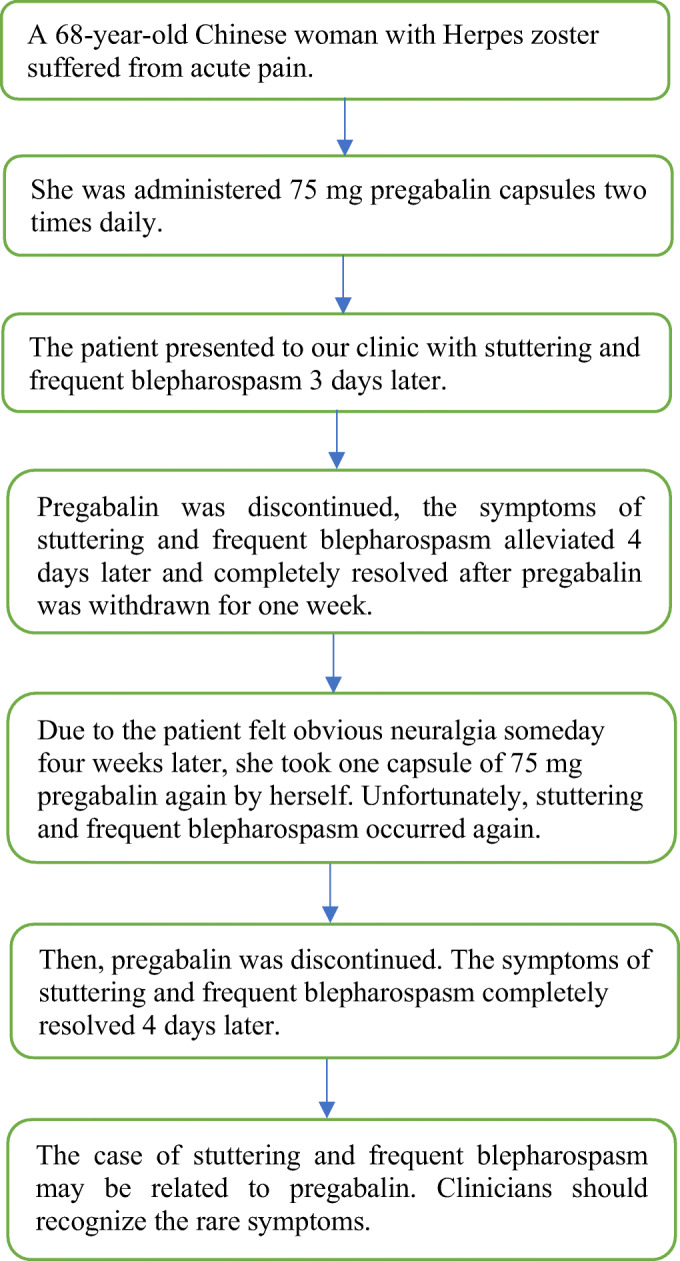
.

.

## Introduction

Herpes zoster (HZ) is an acute, painful, herpes skin disease caused by varicella-zoster virus, which may cause viral meningitis. An important feature of HZ is rash-associated localized pain [1]. The extreme pain of HZ often drives the patient to take analgesic drugs including the alpha-2 delta ligands (pregabalin and gabapentin). It has been shown to be efficacious in the treatment of herpetic pain in patients with HZ [2]. However, the side effects of pregabalin are noteworthy to recognize due to its a wide range of uses. Common side effects of pregabalin reported in the published literature include peripheral edema, unsteadiness, weight gain, liver failure, altered consciousness, heart failure and rhabdomyolysis [3, 4].

Here, we describe a case of stuttering and frequent blepharospasm after pregabalin treatment. To the best of our knowledge, only one report of the patient with stuttering associated with pregabalin has been described in PubMed [5]. Our patient has not only the symptom of stuttering, but also the special clinical characteristic of frequent blepharospasm.

## Reason for report

The main reason for reporting the case study is that the symptoms of stuttering and frequent blepharospasm may be related to pregabalin. That is rare and unusual. Clinicians should be alert to the rare symptoms associated with the use of pregabalin.

## Case presentation

A 68-year-old Chinese woman suffered from burning pain in the skin of left lower limb and followed the skin eruption 2 days later. The eruptions initially presented as papules and plaques of erythema in the dermatome, and the plaques develop blisters within several hours. The lesions continued to appear for several days and reached partially confluence in the district with severe pain. She visited our department and complained about the lesions of clustered blisters and acute pain. Doctors diagnosed her with HZ by differential diagnosis. Our patient was administered 800 mg of aciclovir chewable tablets (Shandong Zibo Xinhua Pharmaceutical Company Limited, Shandong, People’s Republic of China) five times daily for 2 weeks, 75 mg pregabalin capsules (Pfizer Pharmaceuticals Limited, New York, USA) two times daily.

To our surprise, the patient presented to our clinic with stuttering and frequent blepharospasm 3 days later. The patient had no additional signs or symptoms such as peripheral edema, unsteadiness, dizziness. She had a history of periarthritis of shoulder in the past. She felt pain in the left shoulder for several years. The pain did not decrease at rest but increases during activity. She denied taking any other medications. Related laboratory examination was carried out. White blood cell count of 6.28 × 10^9^/l (normal: 3.5–9.5 × 10^9^/l) with 0.55 × 10^9^/l neutrophils (normal: 0.40–0.75 × 10^9^/l); **normal urine color, 6.5 PH level** (normal:4.5–8.0); normal liver enzyme levels of 15.6 U/l AST and 19.10 U/l ALT (normal: 9–50 U/l, 15–40 U/l); normal electrolytes concentration of 4.23 mmol/l potassium (normal: 3.5–5.3/mmol), 142 mmol/l sodium (normal: 137–147/mmol), 102 mmol/l chlorine (normal: 99–110/mmol), 2.22 mmol/l calcium(normal: 2.19–2.54/mmol), 0.96 mmol/l magnesium (normal: 0.70–1.10/mmol). Neurological examination was normal. Magnetic resonance imaging of left shoulder shows that the gap between the humerus and scapula shrinking and the capsular ligament adhesion.

Pregabalin may cause or aggravate myoclonus, ataxia and other adverse events [6–8], and that is why we think that her unusual symptoms may be related to pregabalin. Pregabalin was discontinued, but aciclovir remained. We encouraged the patient to drink more water and the symptoms of stuttering and frequent blepharospasm alleviated 4 days later and completely resolved without any special treatment after pregabalin was withdrawn for 1 week.

Due to the patient felt obvious neuralgia someday 4 weeks later, she took one capsule of 75 mg pregabalin again by herself. Unfortunately, stuttering and frequent blepharospasm occurred again, and then, pregabalin was discontinued. The symptoms of stuttering and frequent blepharospasm completely resolved 4 days later, and the neuralgia subsided after 4 months. No residual symptoms were found at a 6-month follow-up.

## Discussion

Pregabalin is the (S) enantiomer of 3-(aminomethyl)-5-methylhexanoic acid [9]. It binds potently to the alpha-2 delta subunit, reducing calcium influx at nerve terminals, thereby, it reduces the release of glutamate, noradrenaline, and substance P [10]. Now pregabalin has been proven to be effective in the treatment of the neuropathic pain, just as in trigeminal neuralgia [11], and diabetic peripheral neuropathy [12].

Acute HZ and its complication postherpetic neuralgia (PHN) represent a significant challenge to the physicians, 11.9–14.3% of patients will develop PHN following an attack of HZ [13]. Pregabalin was approved by FDA in the treatment of HZ neuralgia in December 2004. Randomized controlled trials have shown that pregabalin not only attenuates preoperative anxiety and pain symptoms, but also improves sleep quality [14]. Given its efficacy in treating pain, pregabalin has been used more widely in patients with HZ neuralgia. But some side effects are gradually recognized [4].

Stuttering is a rare adverse reaction and to the authors’ knowledge, only one previous case was reported in PubMed by Giray et al. in 2016 [5]. In their report, a 31-year-old female patient was assigned a diagnosis of complex regional pain syndrome. After taking of 75 mg pregabalin twice on the first day of treatment, her speech became stuttering. The patient had no additional signs or symptoms except stuttering. She had no comorbid medical illness. One week after discontinuing pregabalin, the stuttering resolved completely. There are some differences or similarities between our patient and the patient reported. They were otherwise healthy, without abnormal laboratory tests, and the same frequency of routine administration. However, our patient presented as not only stuttering but also frequent blepharospasm, and the mechanism of frequent blepharospasm caused by pregabalin is still unknown. And due to the lack of further research, we were unable to determine whether stuttering and frequent blepharospasm was dose dependent.

People usually blink 15–20 times per minute under normal conditions, but frequent blepharospasm is a pathological phenomenon. The ophthalmological and neurological consultation revealed that our patient had no abnormalities except for blepharospasm. Our patient showed bilateral and symmetric blepharospasm that affected her daily activities. After benign essential blepharospasm was ruled out (No ophthalmologic disorders, mental stress, fatigue and others), she was diagnosed with blepharospasm associated with therapy.

Although her stuttering and frequent blepharospasm were associated with the treatment of pregabalin and aciclovir, the symptoms of stuttering and frequent blepharospasm completely resolved without any special treatment after pregabalin was withdrawn for 1 week. Therefore, we believe that pregabalin was probably responsible for her stuttering and frequent blepharospasm. Besides, we use the Naranjo scale to determine whether the adverse drug reaction is associated with the drug use rather than other factors. The drug scored 8 points on the Naranjo scale indicating that the symptom was a “probable” side effect (Table 1).

**Table 1:** The correlation between adverse drug reactions and drug use measured by the Naranjo scale

Naranjo scale for our case	
**Axis**	**Numerical score**
Previous reports on the reaction	1
Temporal illegibility in the onset of the reaction	2
Improvement after drug withdrawal	1
Positive re-challenge	2
Exclusion of alternative causes for the ADR	2
Placebo response	0
Drug concentration and monitoring	0
Dose relationship	0
Previous exposure and cross reactivity	0
Presence of any objective evidence	0
Total score	8
Results: > or = 9 definitive; 5–8 probable; 1–4 possible; < or = 0 unlikely	

Studies show that the occurrence of stuttering is related to the left medial premotor cortex and dopaminergic hyperactivity within CNS [5, 15]. It is possible that pregabalin-related stuttering might cause the imbalance between excitatory and inhibitory neurotransmission leading to dysfunction in white matter fiber tracts. Blepharospasm qualifies as a type of focal dystonia, and the site of pathology in blepharospasm remains unknown. It is possible pathogenesis that increased nigrocollicular pathway activity, and an impairment in corticosensory processing, which possibly lead to a defect of the sensorimotor gating mechanism reduced inhibition of spontaneous blinking [16, 17].

### Outcome

The symptoms of stuttering and frequent blepharospasm completely resolved without any special treatment after pregabalin was discontinued for 1 week.

## Conclusion

The case of stuttering and frequent blepharospasm may be related to pregabalin. We believe such a case is unique because no case of pregabalin-induced frequent blepharospasm has been reported. Clinicians should recognize the rare symptoms.

## Abbreviations

HZ: Herpes zoster
PHN: Postherpetic neuralgia.
